# Occurrence and Distribution of Antibiotics and Antibiotic Resistance Genes in the Water and Sediments of Reservoir-Based Drinking Water Sources in Henan, China

**DOI:** 10.3390/microorganisms13122828

**Published:** 2025-12-12

**Authors:** Wei Yuan, Yijun Shang, Meng Bai, Mingwang Sun, Ziqiang Su, Xi Yang, Luqman Riaz, Yiping Guo, Jianhong Lu

**Affiliations:** 1 School of Ecology and Environment, North China University of Water Resources and Electric Power, Zhengzhou 450046, China; yuanhjaj@163.com (W.Y.); 15039110310@163.com (Y.S.); 15639633289@163.com (M.B.); 17698297136@163.com (M.S.); s15947959957@163.com (Z.S.); yangxi_xiyang@163.com (X.Y.); 2 Department of Environmental Sciences, Kohsar University Murree, Punjab 47150, Pakistan; yuanwei@ncwu.edu.cn

**Keywords:** environmental factors, antibiotic resistance genes, river-sourced reservoirs

## Abstract

The improper use of antibiotics accelerates the emergence of resistance via environmental selection pressures, jeopardizing public health and ecosystems by promoting the worldwide dissemination of antibiotic resistance genes (ARGs). Reservoirs, as crucial water supplies, have been recognized as primary reservoirs of ARGs, particularly those that originate from the Yellow River, necessitating further investigation. This study analyzed 9 ARGs, 3 mobile genetic elements (MGEs), 16 antibiotics, and 10 heavy metals in water/sediments from three reservoirs originating from the Yellow River in Henan Province, China. The findings indicated that antibiotic concentrations in water exceeded those in sediment, with quinolones detected at 100% frequency (5.47–116.03 ng/L) and enrofloxacin predominating (3.36–107.71 ng/L). Redundancy analysis revealed that MGEs exert greater control over ARG dissemination than antibiotics, with intI1 showing strong positive correlations with sul1 (*p* < 0.05). Conversely, heavy metals (Zn, As, Cd) suppress ARG proliferation through negative selection pressures. A network study indicated Mycobacterium, Pseudarthrobacter, and Massilia as critical hosts for *ermB*, *tetA*, and *qnrA*, respectively. Of the three reservoirs, Jian’gang Reservoir, driven by synergistic effects of unique microbial ecology, water self-purification capacity, and flow dynamics, exhibited the best removal effectiveness of ARGs from input to outflow, with 71.75% in the water and 97.91% in the sediment. These findings provide critical insights into the prevalence, migration, and self-purification processes of ARGs in reservoirs originating from the Yellow River, integrating environmental factors and microbial data to clarify the complex dynamics affecting ARG behavior and inform targeted pollution control strategies.

## 1. Introduction

In the 21st century, antibiotic resistance is considered one of the primary challenges to global public health. Antibiotics are often used to prevent and treat diseases; nevertheless, the release of unabsorbed antibiotics into the environment via several pathways may result from the improper and excessive use of antibiotics in human healthcare, livestock management, and agricultural operations [1]. Antibiotics in the environment may exert selection pressures, and in reaction to this antimicrobial selection pressures, bacteria may augment their adaptability by obtaining supplementary genes and expressing resistance genes, then spreading these genes to other bacterial species [2]. This method promotes the global spread of antibiotic resistance, resulting in considerable ecological and public health issues [3]. The presently acknowledged antibiotic-degradation routes include photolysis, chemical transformation, and microbiological catabolism. Photolysis prevails in surface waters and is induced by solar radiation; it occurs via direct photolysis, where the antibiotic molecule absorbs a photon, leading to bond cleavage, or through indirect photolysis facilitated by photochemically generated reactive species such as •OH, ^1^O_2_, and O_2_•^−^. The effectiveness is determined by the compound-specific light-absorption characteristics of the antibiotic and by ambient conditions [4]. Chemical degradation includes hydrolysis, redox, and nucleophilic substitution processes, with speeds significantly influenced by water chemistry (pH, temperature, ionic strength, and oxidant availability); the pH dependency of hydrolysis differs across various antibiotics [5]. Microbial degradation is performed by aquatic microorganisms (bacteria, fungi, actinomycetes) that either use the antibiotic as a carbon and/or nitrogen source or systematically break the molecule by enzymatic catalysis, eventually mineralizing it to CO_2_ and H_2_O. Clarifying these pathways is crucial for comprehending the concentration of profiles and varying outcomes of antibiotics in various aquatic habitats [6].

The pervasive presence of antibiotic resistance genes (ARGs) in the environment is a significant “One Health” concern. The security of human health is threatened by the emergence, evolution, and spread of antibiotic resistance and antibiotic resistance genes (ARGs), as well as the frequency of diseases arising from interactions among humans, animals, and ecosystems [7]. At present, wastewater treatment facilities [8], rivers [9,10], agricultural soils [11], and animal dung are regarded as significant reservoirs of antibiotic-resistant bacteria (ARB), antibiotic resistance genes (ARGs), microbial communities, and mobile genetic elements (MGEs). On one hand, ARGs in the environment may be conveyed to crops via the food chain or irrigation water, or they may directly infiltrate soil and groundwater [12], therefore exacerbating environmental contamination. Antibiotic resistance genes (ARGs) may be transmitted across various bacteria and even between species by horizontal gene transfer and other methods, facilitated by mobile genetic elements (MGEs) [13]. Antibiotic resistance genes (ARGs), quantified using qPCR, vary from antibiotic-resistant bacteria (ARB), which are identified post-cultivation. The former denotes a quantification of certain resistant nucleotide sequences, indicating the potential for resistance. It can swiftly detect genes found in low-abundance or non-culturable microorganisms, including DNA from deceased cells or free DNA, but it cannot ascertain if the genes are expressed or whether the bacteria are alive. Nonetheless, the latter entails the isolation of viable bacteria capable of proliferating on antibiotic-laden media, thereby directly demonstrating “functional resistance,” which is clinically pertinent and linked to the hazards posed by live bacteria; however, it is restricted to the detection of only culturable bacteria [14,15].

Reservoir-type water sources are the primary providers of potable water for residents in aquatic environments and are closely associated with human health [16]. The quality of water in these reservoirs directly affects human health. Multiple studies have verified the presence of antibiotic resistance genes (ARGs) and residual antibiotics in potable water. However, these contaminants are not completely eliminated by the current water treatment technologies [17]. Consequently, it is essential to ascertain the occurrence characteristics and potential diffusion hazards of antibiotics and antibiotic resistance genes in the aquatic habitats of reservoirs promptly.

In addition to antibiotics, recent research suggests that the frequency of antibiotic resistance genes (ARGs) may be influenced by heavy metals [18]. Agricultural practices, industrial activities, and manufacturing processes are the primary sources of heavy metal pollution, prevalent in the environment. Persistence and bioaccumulation exert extended selection pressure on bacteria, promoting the proliferation of mobile genetic elements and antibiotic resistance [19]. Moreover, it alters microbial enzyme activity and metabolic diversity, thereby disturbing ecological equilibrium and reshaping microbial community structures [20]. Moreover, current research indicates that hydrodynamic circumstances significantly affect variations in the abundance of ARGs. For example, suitably prolonging the hydraulic retention time enables the retention and prolonged exposure of host cells harboring ARGs to photolysis, hence, improving the removal effectiveness of ARGs in reservoirs [21,22].

This research examines three drinking water reservoirs in Henan Province supplied by Yellow River water. The study seeks to (1) examine the content and distribution of antibiotics and heavy metals in water and sediment samples from each reservoir, recognizing the distinct biological settings of the reservoirs. (2) Analyze the distribution, abundance, and variability patterns of ARGs and MGEs in the samples. (3) Analyze the makeup of the microbial community in refuse and water to identify potential host bacteria for antibiotic resistance genes (ARGs). (4) Investigate the interconnections between environmental factors, antibiotics, heavy metals, microbial communities, mobile genetic elements, and antibiotic resistance genes, and ascertain the determinants of antibiotic resistance genes. The objective is to elucidate the prevalence of ARGs in reservoir water and sediment, therefore providing essential insights for the protection of reservoir-type water sources, enhancement of reservoir management, and mitigation of hazards associated with the spread of ARGs.

## 2. Materials and Methods

### 2.1. Description of Sampling Sites and Sample Collection

To evaluate the distribution and relation of antibiotics, heavy metals and antibiotic resistance genes in different reservoir-type drinking water sources, Henan Province (Figure S1). Three different types of reservoirs were selected (Linqi Reservoir in Shangqiu, Jian’gang Reservoir in Zhengzhou, Heigangkou Reservoir in Kaifeng), and samples (LQ1, LQ2, JG1, JG2, HGK1, HGK2) were obtained from the water inlet and outlet of the three reservoirs using a coupled “point-belt” gradient sampling method. The detailed sampling protocol is described in Text S1. The corresponding surface water was used to clean the water intake device, and the water samples were collected in 1 L samples at a depth of 10 cm from the water surface, taken 5 times at an interval of 1 h. Each of the five composite water samples was put into a separate sterile water intake bag, stored at 4 °C in an incubator away from light, and sent to the laboratory for basic physicochemical parameters and antibiotic concentration within 24 h, and no longer than 48 h. The remaining water samples were stored in the −80 °C refrigerator.

### 2.2. Determination of Antibiotics and Environmental Variables

The extraction and quantification of tetracyclines (oxytetracycline, OTC; chlortetracycline, CTC; tetracycline, TC), fluoroquinolones (enrofloxacin, EFX; lomefloxacin, LFX), sulfonamides (trimethoprim, TMP; sulfadiazine, SDZ; sulfamerazine, SMZ; sulfamethoxazole, SMX), and macrolides (azithromycin, AZI; clarithromycin, CLA; roxithromycin, ROX) were quantified by LC-MS/MS. After pre-treatment, each solid sample (5 g) or liquid sample (1 L) was enriched and cleaned on a BKMAMLAB HLB cartridge (200 mg/6 mL) preconditioned sequentially with methanol and acetonitrile. Target antibiotics were quantified by triple-quadrupole liquid chromatography–mass spectrometry (TQ-LC-MS); detailed procedures are provided in Text S2 and Table S1. All other physicochemical parameters were determined according to the relevant Chinese national standards (GB 3838-2002 [23]).

### 2.3. Heavy Metals Determination

The concentration of heavy metals in the sediment samples was determined by ICP-OES. The sediment samples were first dried extensively and then processed into fine powders to ensure consistency. Subsequently, 0.3 g of the substance was placed into a 50 mL digestive tube and rehydrated with distilled water. The digestion procedure was executed in compliance with the operating protocol of the fully automated graphite digestion system. Following digestion, the material was diluted to 25 mL using a 2% nitric acid solution. The solution was, thereafter, stirred well and allowed to settle. The supernatant was then transferred for ICP-OES analysis. The detailed analytical methods followed were provided in Text S3 of the Supplementary Materials.

### 2.4. DNA Extraction

All the water samples (1 L) were filtered through 0.45 μm and 0.22 μm pore-size membranes; subsequently, the filter membranes were cut and placed into 1.5 mL centrifuge tubes for DNA extraction. The final DNA extraction solution was used for the antibiotic resistance genes experiment and microbial community analysis. The Ezup Column Soil DNA Purification Kit was used and purchased from Sangon Biotech Co., Ltd. (Shanghai, China). All the DNA samples were stored at −20 °C before using

### 2.5. Quantification of ARGs and MGEs

Nine frequently detected ARGs (*sul1*, *aadA*, *aac(6′)-Ib*, *tetA*, *tetX*, *bla_CTX-M_*, *mphB*, *ermB*, *qnrA*), two integrase genes (*intI1* and *intI2*), one plasmid (*ISCR1*), and the 16S rRNA gene were quantified in the collected samples. The target gene information is shown in Table S2. The gene fragments were synthesized by Sangon Biotech Co., Ltd. (Shanghai, China). The quantitative PCR reactions were performed by the Applied Biosystems QuantStudioTM5 (Thermo Fisher Scientific, Waltham, MA, USA). Following the manufacturer’s instructions, Novo Start^®^SYBR qPCR Super Mix Plus (Novoprotein, Suzhou, China) was used for qPCR amplification of the extracted DNA. The standard curve of selected genes for qPCR is listed in Table S3.

### 2.6. Bacterial Community Analysis

The PCR primers 338F (5′-ACTCCTACGGGAGGCAGCA-3′) and 806R (5′-GGACTACHVGGGTWTCTAAT-3′), which target the V3-V4 region of the bacterial 16S rRNA gene, were used for microbial community analysis. The combined DNA samples were sent to Biomarker Technologies (Beijing, China) for small-fragment library construction and pair-end sequencing using the Illumina HiSeq 2500 (2 × 250 paired ends, Illumina, San Diego, CA, USA) sequencing system. The raw sequencing data were in the FASTQ format. Paired-end reads from the raw DNA fragments were assembled using FLASH (version 1.2.7) [24], then filtered using the Trimmomatic (version 0.33) and UCHIME (version 4.2) software packages to remove low-quality and chimeric sequences [25]. Subsequently, operational taxonomic units (OTUs) were clustered using the UCLUST software within QIIME (version 1.8.0), and OTUs with abundances below 0.03% were removed. Taxonomic classification was carried out in each sample using the RDP (Ribosomal Database Project) Classifier [26] with a bootstrap cutoff value of 50% to distinguish the taxonomic level of the sequence and the GreenGene database [27] to identify the 16S genes.

### 2.7. Data Analysis

All statistical analyses were conducted using IBM SPSS 28 Graphics (Statistical Graphics Corp., Princeton, NJ, USA) and GraphPad Prism 8 (GraphPad Software, La Jolla, CA, USA). Detrended correspondence analysis (DCA) was used to determine the length of the ARGs gradient composition [28]. In accordance with DCA recommendations, redundancy analysis (RDA) was performed with Canoco 5.0 software (Microcomputer Power, Berkeley, CA, USA). OriginPro 2024 was utilized to generate histograms and line charts illustrating the abundance of bacteria at the phylum level, along with the absolute and relative abundance of antibiotic resistance genes (ARGs). Network analyses utilizing Spearman analysis were conducted with IBM SPSS, while Gephi (version 0.9.2) served as the visualization platform.

## 3. Results and Discussion

### 3.1. Environmental Variables and Antibiotics in the Water and Sediments

#### 3.1.1. Environmental Variables in the Water

**Figure 1:**
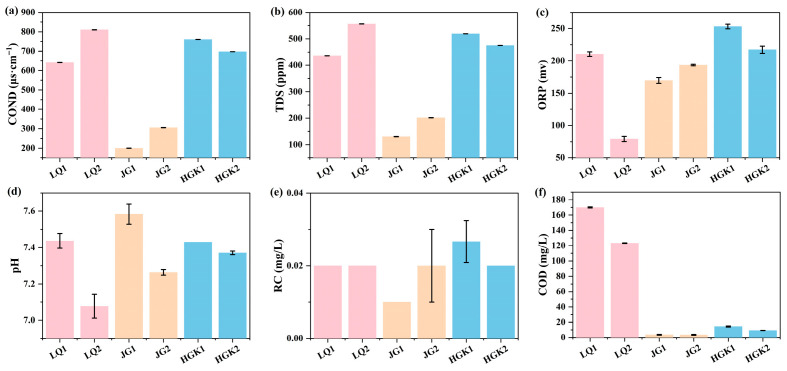
In situ measurement of environmental variables at the inlet and outlet of reservoirs: (**a**) COND; (**b**) TDS; (**c**) ORP; (**d**) pH; (**e**) RC; (**f**) COD. (1 represents the reservoir inlet; 2 represents the reservoir outlet).

Figure 1 and Table S4 provide the in situ characteristics of several lakes, including conductivity (COND), total dissolved solids (TDS), oxidation–reduction potential (ORP), residual chlorine (RC), pH, and chemical oxygen demand (COD). At each sampling location, basic physicochemical properties of the water samples were assessed using three parallel replicates, measured concurrently according to the standard techniques, with the arithmetic mean serving as the final result. The average conductivity values in JG Reservoir (from 200.03 ± 0.57 to 329.83 ± 0.12 μs·cm^−1^) were lower than LQ and HGK Reservoir (from 641.00 ± 0.36 to 810.00 ± 0.17 μs·cm^−1^), and the values of TDS exhibited a comparable trend. The levels of ORP, RC, and pH were rather consistent in the observed reservoirs. The maximum amounts of COD reached 146.61 ± 0.53 mg/L in LQ, followed by HGK and JG Reservoirs. The COD content exhibited a progressive decline from the inlet to the exit, and the water quality metrics of the effluent from the three reservoirs were significantly superior to those of the entering water.

#### 3.1.2. Antibiotic Concentration in the Water and Sediments

**Figure 2:**
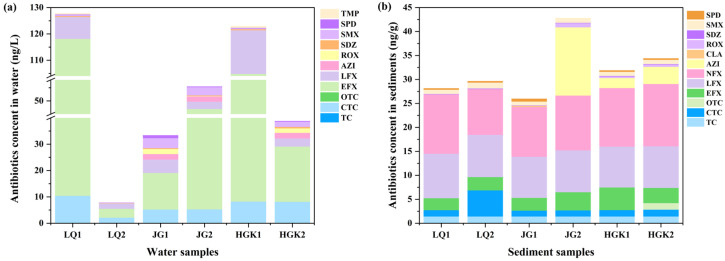
Variations in antibiotic concentrations in the water (**a**) and sediments (**b**) at the inlets (1) and outlets (2) of three reservoirs.

Figure 2 and Tables S5 and S6 show the different types of antibiotics found in the water and sediments, as well as their corresponding concentrations. In the LQ Reservoir, CTC and quinolone antibiotics were detected at 33.3% and 100%, respectively. The reservoir also demonstrated excellent removal efficiency for CTC, EFX, and LFX, with removal rates of 79.63% (10.41–2.12 ng/L), 96.88% (107.71–3.36 ng/L), and 74.64% (8.32–2.11 ng/L), respectively. EFX was reduced by 78.28% (96.58–20.98 ng/L) in the HGK Reservoir. Nonetheless, in the JG Reservoir, EFX was increased, which might be attributed to point-source pollution in this area. Except for EFX, the concentration fluctuation patterns of CTC and LFX were identical in the JG and HGK reservoirs. In the JG Reservoir, LFX degraded by 45.60%, but in the HGK Reservoir, it degraded by 81.41%. CTC concentrations were almost consistent at the reservoirs’ inflow and departure.

Tetracycline antibiotics (TCs) and fluoroquinolones (FQs) were identified in water samples from all three reservoirs. Among all identified antibiotic categories, enrofloxacin (EFX) had the greatest concentration (3.36–107.71 ng/L). Unlike the data indicating the highest concentration of sulfonamide antibiotics in the Danjiangkou Reservoir [29], the total concentration of fluoroquinolone antibiotics ranged from 5.47 to 116.03 ng/L. Fluoroquinolones are the most often prescribed antibiotics in aquaculture; hence, their elevated concentration may signify their use in human activities, including aquaculture inside the reservoir [30]. Moreover, fluoroquinolones are often used in livestock management. Specifically, small domestic farms located near the reservoir, which exhibit insufficient biosecurity measures, routinely give enrofloxacin over extended durations, thus elevating the total instances of enrofloxacin exposure occurrences [31]. Mismanaged manure and wastewater may infiltrate rivers via sewage discharge, precipitation runoff, and surface runoff, ultimately contaminating reservoirs. CTC was identified in quantities between 2.12 and 10.41 ng/L, ranking just below fluoroquinolones. Tetracyclines have a lower overall concentration compared to fluoroquinolones, and there has been less research into their occurrence in natural water bodies. The concentrations of macrolides and sulfonamide antibiotics in the three reservoirs are rather low. This may be due to their reduced occurrence in proximate human activities or to the self-purification mechanisms of aquatic systems, which may expedite the degradation processes of these two classes of antibiotics in the environment.

The categories, ratios, and concentration trends of residual antibiotics in the sediments of the three reservoirs followed patterns identical to those seen in the water. Figure 2b shows the distribution of antibiotics in sediments. Antibiotic concentrations in the water column were much greater than in the sediments. The progressive buildup of antibiotics in the sediments, resulting from sediment adsorption and their eventual movement toward the reservoir outflow as water flowed, illustrated the distinctions between the two. EFX values in sediments varied from 2.48 to 4.71 ng/g and exhibited no significant difference between the inflow and outflow locations of the same reservoir. AZI levels were stable in the sediments of the HGK Reservoir, but they exhibited significant enrichment in the JG reservoir, increasing from ND to 14.23 ng/g. We deduce that wastewater containing AZI is released into the middle-lower sections of the JG reservoir. Such inputs not only suppress the activity of native bacteria but also, due to sediments absorbing much less sunlight than surface water, concurrently diminish both biodegradation and photolysis, eventually resulting in AZI buildup at site JG2. The increase in CTC at location LQ2 may also be attributed to the simultaneous decline in the two primary degradation routes. TC was absent in the water bodies of the three reservoirs, although it was detected in the sediments at a comparable concentration (1.41 ng/g). This may relate to the slightly alkaline characteristics of water bodies, which might facilitate the precipitation of tetracycline antibiotics, enabling their adsorption in sediments [32]. Fluoroquinolone antibiotics were identified in all soil samples, exhibiting greater quantity than other antibiotics, and the species detected surpassed those found in the water. This finding aligns with previous research indicating that tetracyclines (TCs) and fluoroquinolones (FQs) were present in greater concentrations in sediment than in water in the Chaobai River [33]. This phenomenon is likely attributable to the hydrophobic nature of TCs and FQs, making them prone to photolysis and sediment adsorption [34]. Moreover, the reduced degradation rate of antibiotics by bacteria in sediment enhances their persistence in this medium [35]. The detection rate and concentration range of sulfonamide antibiotics in reservoir sediments were inferior to those in the water column, likely due to the hydrophilic characteristics of sulfonamides, which enable their persistence in the aqueous phase. In comparison to TCs and FQs, SAs and MLs antibiotics were used less in agriculture and animal husbandry. Inadequate stability, vulnerability to microbial degradation, and interactions of degradation products with other compounds may all contribute to the antibiotics’ diminished concentrations [36,37].

### 3.2. Heavy Metals Concentration in the Sediment

**Figure 3:**
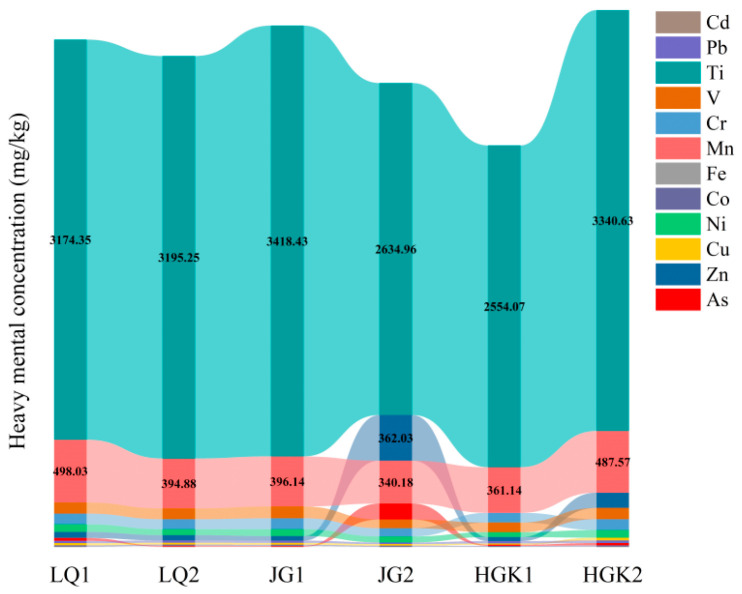
Heavy metal concentration in the sediments.

Figure 3 and Table S7 show the distribution of heavy metal concentrations in reservoir sediments. The accumulation and concentration levels of these metals, in decreasing order, are as follows. The average amounts of heavy metals at each sediment sample location are listed below: Ti (2554.07–3418.43 mg/kg) > Mn (340.18–498.03 mg/kg) > Zn (31.21–362.03 mg/kg) > V (68.62–93.93 mg/kg) > Cr (65.59–87.42 mg/kg) > Ni (40.73–61.68 mg/kg) > As (5.53–126.10 mg/kg) > Pb (13.16–20.38 mg/kg) > Cu (10.50–21.37 mg/kg) > Co (7.18–9.94 mg/kg). There were no significant differences in heavy metal concentrations across the reservoir sediment sample sites. The highest concentrations of Ti were found, perhaps due to its natural abundance and occurrence in a variety of minerals. Natural processes may promote the migration of titanium from minerals to soil and sediments, resulting in higher concentrations [38]. The lower amounts of As, Fe, and Co in the three reservoir sediments might be attributed to their restricted adsorption capacity and accumulation rates [39]. Zn concentrations were much higher in sediments near the JG Reservoir outflow (JG2). This surge might be attributed to the outflow of zinc-laden wastewater or agricultural operations in the vicinity, which could result in the release of Zn into the water and sediments. Agricultural activities contribute significantly to the entry of Zn into sediments [40]. Heavy metals have several sources. The continuous use of organic fertilizers and pesticides in agriculture, along with sewage irrigation, may result in the accumulation of certain heavy metals [41].

### 3.3. Abundance and Diversity of ARGs and MGEs

**Figure 4:**
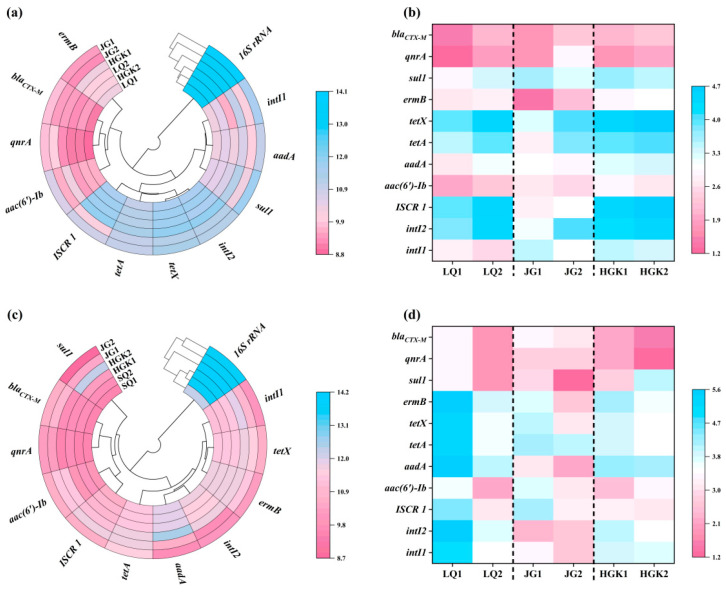
(**a**,**b**) Absolute and relative abundance of ARGs, MGEs, and 16S rRNA in the water; (**c**,**d**) Absolute and relative abundance of ARGs, MGEs, and 16S rRNA in the sediment.

This study identified the presence of three prevalent integrase genes (*intI1*, *intI2*, and *ISCR1*) within the samples, as well as eight common antibiotic resistance genes (ARGs): *tetA*, *tetX*, *aac(6′)-Ib*, *aadA*, *sul1*, *ermB*, and *bla_CTX-M_*, which confer resistance to tetracyclines, aminoglycosides, sulfonamides, macrolides, and β-lactam antibiotics. Figure 4 illustrates the absolute abundances of mobile genetic elements (MGEs) and antibiotic resistance genes (ARGs) in the water and sediments of several reservoirs, together with their corresponding relative abundances normalized to the 16S rRNA gene.

Figure 4a illustrates the absolute abundances of 16S rRNA, MGEs, and ARGs in the water of the three reservoirs. The bacterial 16S gene copy counts varied from 1.19 × 10^7^ copies/L to 1.18 × 10^8^ copies/L, with the peak abundance of 16S detected in the entrance of the JG Reservoir. The abundance of 16S showed a declining trend from the inlets to the outflows of the three reservoirs. The research revealed that the absolute abundance ranges of the *intI1*, *intI2*, and *ISCR1* integrase genes were 5.03 × 10^3^ copies/L to 4.02 × 10^5^ copies/L, 1.54 × 10^5^ copies/L to 6.54 × 10^5^ copies/L, and 1.31 × 10^4^ copies/L to 8.64 × 10^5^ copies/L, respectively; furthermore, significant variations were noted across different sampling locations. In this work, the integrase gene *intI2*, serving as an indicator for the horizontal gene transfer of antibiotic resistance genes (ARGs) [42], fecal contamination, or anthropogenic influence [43], was shown to be more prevalent than *intI1* and *ISCR1*. Of the six identified ARGs, it is significant that tetracycline resistance genes exhibited the greatest abundance, ranging from 2.46 × 10^5^ to 1.12 × 10^6^ copies/L. At the entrance of JG Reservoir, tetracycline resistance genes constituted just 28.56% of the total ARGs; however, at other sample locations, their percentage ranged from 82% to 90%, greatly exceeding that of *qnrA*, *bla_CTX-M_*, and *ermB* genes by one to two orders of magnitude. This may be ascribed to the superior selection benefit of tetracycline resistance genes under environmental stressors [44]. In contrast to the 35.62% decrease in the total absolute abundance of antibiotic resistance genes (ARGs) in HGK Reservoir water, ARGs in JG Reservoir water displayed the highest removal efficiency at 71.75%, with aminoglycoside (91.98%) and sulfonamide (95.74%) resistance genes showing notably substantial reductions. In the LQ Reservoir, the overall abundance of ARGs at the exit rose by about 20% relative to the inlet. All categories of resistance genes exhibited a pattern of enrichment in the direction of water flow inside the reservoir. This may be ascribed to the sophisticated aquaculture practices in the upper reaches of LQ Reservoir and the prevalent use of diverse antibiotics by adjacent animal farms, which hindered the efficient reduction in MGEs and ARGs by bacteria and microorganisms. In contrast to chemical pollutants, antibiotic resistance genes (ARGs) may replicate and spread among bacteria by vertical transmission and horizontal gene transfer [45,46], indicating that ARGs persisting in reservoirs may continue to represent a potential risk to human health and ecological integrity.

Figure 4c,d shows the absolute and relative abundances of 16S rRNA, mobile genetic elements (MGEs), and antibiotic resistance genes (ARGs) in sediments from three reservoirs. Sediments contained all ARGs found in the water. The concentration of ARGs in the sediments surpassed that in the water, similar to a prior study [47,48], but was lower than the range found (1.80 × 10^8^–1.80 × 10^11^ copies/g) in the sediments of Qingcaosha Reservoir [49]. The abundance of ARGs in sediments near LQ Reservoir outflow increased somewhat, with the absolute abundance of 16S ranging from 1.84 × 10^6^ to 6.83 × 10^7^ copies per gram. In the JG Reservoir, total ARGs reduced from 1.56 × 10^6^ copies/g to 6.04 × 10^5^ copies/g, a 61.36% decline. All resistance genes were lowered by 97.91%, which is two orders of magnitude. In the HGK Reservoir, the total abundance of ARGs in the exit sediments increased by 246.39% compared to the inlet. The degradation of sediments by water flow, as well as the inflow of sediments from surrounding wetlands, may increase the frequency of antibiotic resistance genes. The levels of *sul1* and *aac(6′)-Ib* increased by two orders of magnitude, reaching 1.74 × 10^4^–1.84 × 10^6^ copies/g and 9.54 × 10^3^ copies/g–3.00 × 10^5^ copies/g, respectively. The substantial change in *sul1* and *aac(6′)-Ib* might be attributed to their genetic link with mobile genetic elements (MGEs), which allow for the fast spread of antibiotic resistance genes (ARGs) across microbial populations via horizontal gene transfer (HGT). Moreover, changes in the diversity and prevalence of MGEs may expedite the dissemination of antibiotic resistance within ecosystems [33,50]. Moreover, the nutrient-dense soil and particle deposition fostered an environment conducive to a diverse microbial community inside the sediments. Alterations in the composition and operation of microbial communities, coupled with anthropogenic activities, significantly influence the distribution of antibiotic resistance genes (ARGs) [51].

### 3.4. Composition of Bacterial Communities

#### 3.4.1. Bacterial Community Richness and Diversity

Table S8 provides estimates of α-diversity, richness, and coverage of microbial communities in water and sediment samples collected at the reservoir sampling site. Sequencing coverage exceeded 0.99 in all samples, with water samples achieving 0.9997 and sediment samples ranging from 0.9989 to 0.9997. The Shannon and Simpson indices analyzed diversity, whereas the Chao1 and Ace indices assessed community richness [52]. When compared to other media, silt samples were much more diverse and varied than water samples. Among the water samples, JG2 had the lowest Shannon, Simpson, Chao1, and Ace indices, suggesting less diversity and richness than the other sampling sites. Bacterial richness and diversity in water samples decreased from reservoir intake to departure. With the exception of a little increase in the Simpson index at the JG Reservoir exit, the bulk of sediment sample values were lower than those at the inlet, with minimal changes. Bacterial richness was substantially higher at the LQ and HGK Reservoir exits than at the entrances, with no significant changes in diversity.

#### 3.4.2. Characteristics of Bacterial Communities at the Phylum and Genus Levels

**Figure 5:**
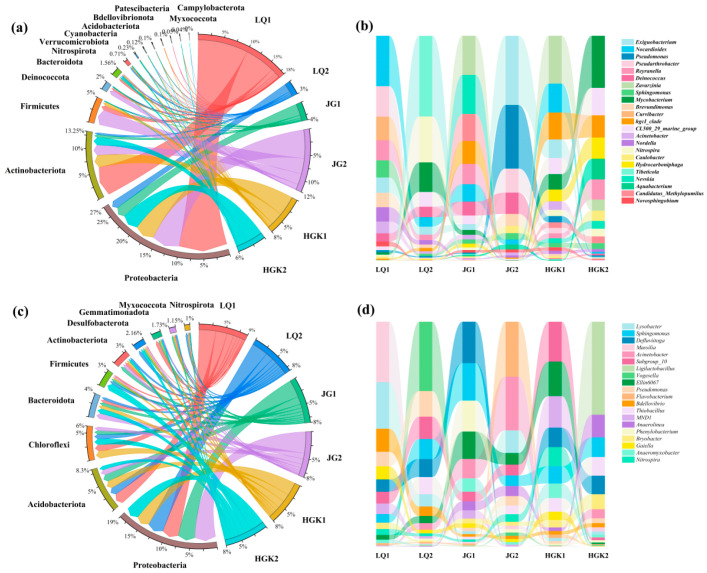
(**a**) Microbial community structure in the water bodies at the phylum level; (**b**) structure of the top 20% abundant bacteria in the water bodies at the genus level; (**c**) the top 20% abundant bacteria in the sediments at the phylum level; (**d**) the top 1% abundant bacteria in the sediments at the genus level.

The microbial community structure was investigated utilizing high-throughput sequencing of the 16S rRNA gene. Figure 5 depicts the absolute abundance of microbial communities in water and sediments from several reservoirs. Furthermore, LEfSe analysis was carried out on the differential species (top 50 in abundance) in the water and sediment of the three reservoirs, as shown in Figures S2 and S3. The microbial community composition differed significantly between each reservoir’s intake and departure. These differences may serve as biological markers for categorization and reveal the selection effects of environmental factors on microbial populations.

Figure 5a,b illustrates the predominant microbial species at different sampling locations inside the water bodies of three reservoirs. Thirteen principal phyla were identified throughout the three reservoirs, including Proteobacteria, Actinobacteriota, Firmicutes, Bacteroidota, and Deinococcota, predominating at all sampling locations. Aside from the HGK Reservoir outflow, where Proteobacteria and Firmicutes were predominant, Proteobacteria and Actinobacteriota ranked as the top and second most prevalent phyla in the water bodies of the other sample locations. The findings aligned with prior research, which recognized Proteobacteria, Actinobacteriota, and Firmicutes as the predominant phyla in freshwater ecosystems and suggested that they might function as the principal carriers of ARGs [53,54]. Both natural processes and human activities may significantly influence differences in microbial community abundance [55]. Research indicates that Actinobacteriota may produce therapeutically relevant antibiotics [56], contributing to their status as the second most prevalent phylum. A total of 216 genera were identified at the genus level. Figure 5b illustrates the prevalent and varied bacterial species identified in the water at the sampling locations. *Nocardioides*, *Pseudarthrobacter*, *Curvibacter*, and *Reyranella* were the predominant taxa at the inflow of the LQ Reservoir; however, their absolute abundance was inferior to that of *Tibeticola*, *Nitrospira*, and *Mycobacterium* at the outflow. The predominant species in the outflow of the JG Reservoir were *Exiguobacterium* and *Pseudomonas*, while *Zavarzinia* and *Nevskia*, which were more prevalent at the inflow, were seldom detected in the outflow water.

Marked disparities in community structure were seen across several sample locations within each reservoir. The total abundance of dominant taxa exhibited considerable variation across different locations within the same reservoir. At the genus level, the absolute abundance of microorganisms at the outflow of LQ Reservoir and HGK Reservoir decreased by 85.78% and 26.29%, respectively, in comparison to the inflow. Conversely, JG Reservoir exhibited a 241.73% enrichment of microbial species from intake to discharge. The data indicate that fluctuations in reservoir conditions, including antibiotic concentrations, may substantially influence microbial populations.

The sediments contain a higher abundance and variety of microorganisms compared to the water. A total of 51 phyla were identified at the phylum level. Figure 5c illustrates the 10 most prevalent phyla according to total absolute abundance in sediments. The five phyla with the largest absolute abundance were Proteobacteria, Acidobacteriota, Chloroflexi, Bacteroidota, and Firmicutes, which corresponded to the dominating phyla identified in the sediments of the Three Gorges Reservoir and Miyun Reservoir [57,58]. Proteobacteria were present at all sampling locations and had the greatest abundance, making them one of the most prevalent taxa in reservoir and lake sediments [59]. Acidobacteriota was the second most prevalent phylum in LQ1, LQ2, JG1, JG2, and HGK1, but Firmicutes was the second most prevalent phylum in HGK2. Proteobacteria, Bacteroidota, and Firmicutes were identified as the predominant phyla in the water and sediments of the three reservoirs. This may be ascribed to their involvement in the metabolic cycling of diverse substances, significantly influencing the reservoir’s total microbial community [37]. No discernible trend was seen in the alterations in microbial community structure at the genus level. Figure 5d illustrates that the predominant taxa differ across various sampling locations. At site LQ1, the predominant genera were *Massilia* (17.11%) and *Lysobacter* (13.22%); at site JG1, *Vogesella* (15.69%); at site JG2, *Defluviitoga* (7.76%) and *Sphingomonas* (7.01%); at site HGK1, *Flavobacterium* (9.28%) and *Acinetobacter* (9.08%); and at site HGK2, *Subgroup_10* (5.97%) and *Ligilactobacillus* (13.68%). The disparities in predominant genera among locations may be ascribed to fluctuations in water and sediment conditions, which affect microbial community composition. *Massilia* is a genus of Gram-negative bacteria (proteobacteria) found in several habitats, including soil and water. It may decompose several environmental contaminants, including aromatic hydrocarbons and insecticides. Certain *Massilia* strains possess integron-mediated antibiotic resistance genes (e.g., *sul1*, *aadA*), potentially facilitating the dissemination of environmental antibiotic resistance [60]. *Sphingomonas*, similar to *Massilia*, is classified under the Proteobacteria genus and has the capability to degrade several environmental organic contaminants [59,61]. *Ligilactobacillus* exhibits resistance to several antibiotics due to its distinctive cell wall composition [62]. With the exception of a few prominent degrading genera (e.g., *Flavobacterium*, *Sphingomonas*, *Acinetobacter*) [63,64,65], the majority of dominant bacterial taxa have not been documented to exhibit antibiotic-degrading abilities, which may significantly contribute to the typically low rates of antibiotic degradation observed in the sediment phase.

The complexity and variety of microbial communities in water and sediments exhibited considerable variation among reservoirs and sampling locations. The predominant phyla among the communities were Proteobacteria, Acidobacteriota, Actinobacteriota, Chloroflexi, Bacteroidota, and Firmicutes. The natural environment of reservoirs and adjacent human activities impact microorganisms, leading to substantial alterations in the bacterial community structure at the genus level in both water and sediments.

### 3.5. Network Analysis Between ARGs and Bacterial Communities

**Figure 6:**
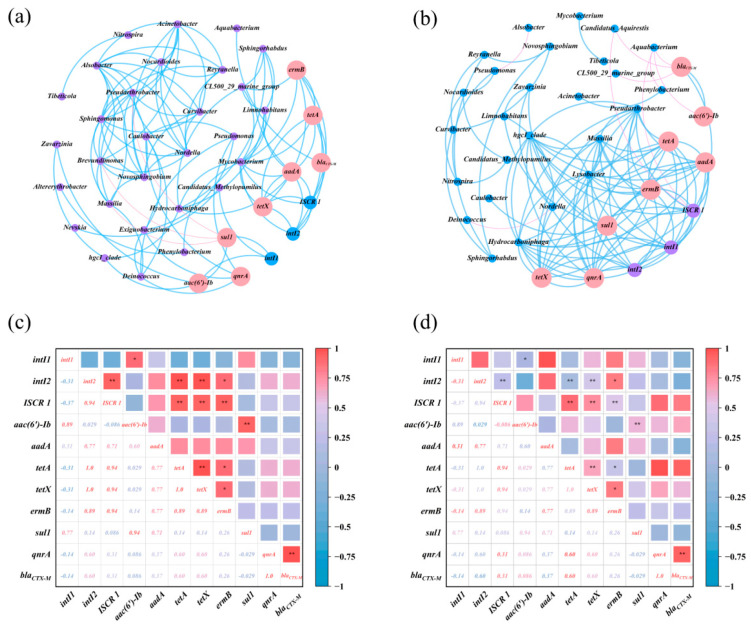
(**a**,**b**) Network relationship maps of microbial communities, MGEs, and ARGs in the water and sediments; (**c**,**d**) heatmaps of the correlation between ARGs in the water and sediments (*, *p* < 0.05; **, *p* < 0.01).

Figure 6 depicts the examination of co-occurrence patterns among microbial communities, mobile genetic elements (MGEs), and antibiotic resistance genes (ARGs) in reservoir water and sediments, as well as the relationship between MGEs and ARGs using Spearman correlation. The water network in Figure 6a had 40 nodes and 105 edges, while the sediment network in Figure 6b had 37 nodes and 107 edges. Antibiotic resistance genes (ARGs) and mobile genetic elements (MGEs) in aquatic environments and sediments showed a robust positive correlation with microbial populations. The intricate connections between bacteria and ARGs suggest a mutual influence. Although possessing fewer nodes, the sediment network attained an equivalent number of edges, leading to a superior average degree and more robust intra-module connections. Conversely, the water-phase network had a somewhat greater number of nodes without a corresponding rise in edges, suggesting a broader diversity of possible hosts or gene types among ARGs, MGEs, and bacterial taxa in the water column.

In aquatic conditions, *intI1* was positively correlated with *aac(6′)-Ib* and *sul1* (*p* < 0.05), whereas *intI2* was positively correlated with *ISCR1*, *aadA*, *tetA*, and *ermB*. The results confirm the co-occurrence of MGEs and ARGs in the samples, which is consistent with previous studies [17]. *aac(6′)-Ib* correlated positively with *sul1*, *ermB* with *tetA* and *tetX*, *aadA* with *tetA* and *tetX*, among the antibiotic resistance genes in the water. *Mycobacterium* showed positive relationships with *ermB*, *tetA*, *tetX*, *aadA*, *qnrA*, *ISCR1*, and *intI2*, suggesting that it might serve as a host for these antibiotic resistance genes and mobile genetic elements. *Pseudarthrobacter* was positively associated with *qnrA*, whereas *sul1* was negatively associated with *Pseudarthrobacter*, *Brevundimonas*, and *Massilia*. Furthermore, *Exiguobacterium* and *Caniobacter*, both Firmicutes, showed a positive connection with *qnrA*. Aside from the aforementioned species, *Hydrocarboniphaga* and *Pseudomonas* had favorable interactions with *aadA* and *qnrA*, respectively. Moreover, β-Proteobacteria and γ-Proteobacteria, the most diverse microbial groupings across the three reservoirs, significantly influenced the variety of ARGs present in the water. Proteobacteria significantly contributed as the most diverse microbial community in river environments [66]. The co-occurrence network analysis of antimicrobial resistance genes (ARGs), mobile genetic elements (MGEs), and probable host microorganisms in aquatic environments significantly facilitates the investigation of ARG prevalence and dissemination.

Figure 6b,d shows that in the sediment ecosystem network and the ARGs correlation heatmap, the selected MGEs (*intI1*, *intI2*, and *ISCR1*) exhibited positive correlations with one another as well as with the ARGs *aadA*, *tetA*, *tetX*, *ermB*, and *qnrA*, reflecting the correlations observed in the water ecosystem and emphasizing the role of MGEs in the proliferation and dissemination of ARGs [67]. Significantly, *bla_CTX-M_* and *aac(6′)-Ib* showed a link with *ISCR 1*, which has potential hosts (*Candidatus_Aquirestis* and *Aquabacterium*) with *bla_CTX-M_*. *Limnohabitans*, *Zavarzinia*, and *Caulobacter* were potential hosts for *sul1*, whereas *Lysobacter* hosted *intI1* and *intI2*. In contrast to water, *Pseudarthrobacter*, *Massilia*, *hgcl_clade*, and *Hydrocarboniphaga* were common hosts for *aadA*, *tetA*, *tetX*, *ermB*, *qnrA*, and all MGEs, suggesting that the MGEs originated from a single source. Prior research has shown that antibiotic resistance genes (ARGs) are often located inside mobile genetic elements (MGEs), which may disseminate across bacterial populations via horizontal gene transfer (HGT). Consequently, MGEs, via prospective microbial hosts, may expedite the dissemination of ARGs in ecosystems, corroborating prior observations [68,69]. Moreover, biochemical interactions between acquired genes and cellular matrix may influence variations in microbial community systems, modifying the horizontal gene transfer of resistance genes [54]. In the sedimentary environment, antibiotic resistance genes (ARGs) are mostly associated with the phyla Proteobacteria and Actinobacteria, namely α-, β-, and γ-proteobacteria. Proteobacteria are significant pathogens; infections caused by ARG-bearing pathogens may complicate treatment and provide environmental health hazards [67]. Moreover, elevated antibiotic concentrations and the presence of heavy metals may expedite the emergence and dissemination of antibiotic resistance genes in bacteria [17].

### 3.6. RDA Between ARGs and Bacterial Communities

Despite the water originating from the Yellow River, variations in environmental parameters and external pollutants (including differences in the types and concentrations of heavy metals and antibiotics) across different reservoirs may significantly impact the prevalence of mobile genetic elements (MGEs) and antibiotic resistance genes (ARGs), consequently influencing the distribution and transfer of ARGs within the water-sediment system.

**Figure 7:**
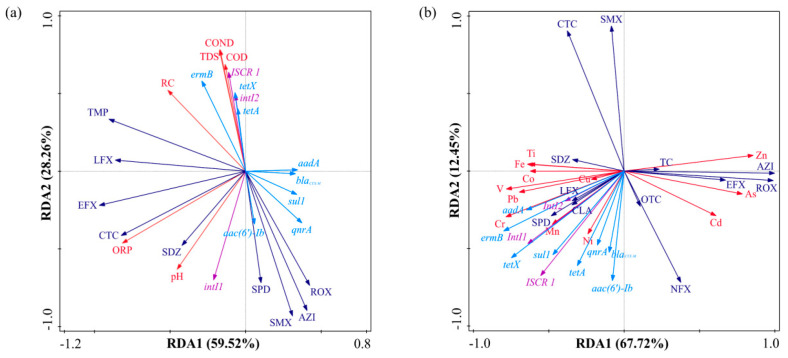
(**a**) Redundancy analysis (RDA) among environmental factors, antibiotics, MGEs, and ARGs in the water; (**b**) RDA among heavy metals, antibiotics, MGEs, and ARGs in the sediments.

Figure 7 depicts a redundancy analysis (RDA) that uses numerous metrics from reservoir water and sediments to separate the contributions of the given factors on the prevalence of ARGs. Figure 7a depicts a redundancy analysis (RDA) of environmental factors, antibiotics, mobile genetic elements (MGEs), and antibiotic resistance genes (ARGs) in the water of three reservoirs. The first two axes combined explained 87.78% of the variation, with RDA1 and RDA2 contributing 59.52% and 28.26%, respectively. Figure 7b shows the RDA for heavy metals, antibiotics, and MGEs in relation to ARGs in the sediments of the three reservoirs. The analysis revealed that the major axes RDA1 and RDA2 accounted for 67.72% and 12.45% of the variation in ARGs and MGEs, respectively.

Mobile genetic elements significantly influence the distribution of antibiotic resistance genes in aquatic ecosystems and sediments. Research indicates that mobile genetic elements (MGEs) are the principal agents of antibiotic resistance gene (ARG) dissemination; their increased prevalence enhances ARG propagation, surpassing the influence of microbial communities [70]. Redundancy analysis (RDA) validates the significance of mobile genetic elements (MGEs) in facilitating the transmission of antibiotic resistance genes (ARGs) via horizontal gene transfer (HGT). MGEs in aquatic habitats have a substantial association with environmental parameters such as TDS, COND, COD, and RC, indicating that the distinct environmental conditions of each reservoir affect the distribution of MGEs, subsequently influencing the distribution of ARGs and host composition [71]. Most heavy metals in sediments, except Cd, As, and Zn, have significant associations with the distribution of ARGs. Toxicity thresholds likely explain this phenomenon: when the activity of toxic ions beyond cellular tolerance limits, heavy metals inhibit both the prevalence and horizontal transmission of antibiotic resistance genes, resulting in negative selection pressure. Heavy metals may influence the dissemination of antibiotic resistance genes (ARGs) via selection pressure, co-resistance, promotion of horizontal gene transfer (HGT), and antibiotic synergy. Antibiotics have a lesser impact on antibiotic resistance genes (ARGs) compared to heavy metals. In the aquatic environment (Figure 7a), all detected medications exhibited a substantial negative correlation with all antibiotic resistance genes (ARGs). The varying concentration patterns of antibiotics and ARGs are likely due to the swift dilution resulting from high-flux discharge at the downstream outlet, which disrupts water exchange and concurrently decreases hydraulic retention time, thereby further hindering antibiotic degradation efficiency in the aqueous phase. A robust positive association exists between *intI1*, oxidation–reduction potential (ORP), and pH levels. Figure 7b illustrates that sulfonamide antibiotics, including SPD, SDZ, CLA, and LFX, had substantial connections with antibiotic resistance genes (ARGs), while other pharmaceuticals displayed negligible impacts in the sediment. This situation partly indicates residual pollution from previous agricultural or veterinary practices, with the potential for continuous environmental contributions. Moreover, heavy metals were mostly accumulated in association with integrons and antibiotic resistance genes (ARGs). The observed disparities may be ascribed to the prolonged accumulation of heavy metals in the environment, which exerts continuous selection pressure on microbial communities inside reservoir sediments. Antibiotics, meanwhile, are often fragile and susceptible to environmental deterioration, leading to diminished concentrations and, hence, exerting less selection pressure on microbial populations.

## 4. Conclusions

The present study thoroughly examined the distribution, transmission, and primary determinants of antibiotic resistance genes (ARGs) in the water and sediments of three reservoirs fed from the Yellow River, which share similar water sources but possess differing surrounding ecosystems. This work demonstrates that mobile genetic elements (MGEs), rather than antibiotics, are the primary facilitators of antibiotic resistance gene (ARG) spread, elucidating that heavy metals indirectly influence ARG abundance by modulating MGEs, and highlighting the significance of essential host bacteria such as Mycobacterium and Pseudarthrobacter. The Jian’gang Reservoir demonstrated superior removal effectiveness of ARGs in sediments compared to water. The enhanced performance in sediments is ascribed to the synergistic effects of distinct microbial ecology, sediment adsorption, and reduced antibiotic degradation rates, which together inhibit the growth of ARGs. Future research may concentrate on the prolonged observation of ARGs migration and transformation within reservoir–river continua, investigating microbial community-based in situ ARGs removal technologies, and incorporating metagenomics to elucidate the mechanisms governing MGEs-mediated horizontal transfer of ARGs. As of now, there is no historical or multi-year continuous monitoring dataset available for the specified three reservoirs and their contiguous sections of the Yellow River. This study intends to address a gap by conducting seasonal sampling campaigns in subsequent research to elucidate the temporal patterns and variations in antibiotic concentrations throughout the reservoir. Furthermore, due to the propensity of specific antibiotics associated with particulate matter, forthcoming analyses will measure total organic carbon (TOC) and particle-size distribution to enhance the interpretation of antibiotic sorption to sediments. These initiatives will collectively improve our comprehension of antibiotic resistance genes (ARGs) and antibiotic dynamics in freshwater ecosystems and facilitate the formulation of targeted mitigation strategies.

## Data Availability

The original contributions presented in this study are included in the article/Supplementary Materials. Further inquiries can be directed to the corresponding author.

## Supplementary Materials

The following supporting information can be downloaded at: https://www.mdpi.com/article/10.3390/microorganisms13122828/s1, Text S1. Detailed design of the sampling methodology; Text S2. The TQ-LC-MS analysis of the antibiotics; Text S3. The ICP-OES analysis of the heavy metals; Figure S1: Geographical distribution of sampling points in reservoir-type water source area; Figure S2: Distribution of the top 50 differentially abundant species in the water bodies of various reservoirs based on LEfSe analysis; Figure S3: Distribution of the top 50 differentially abundant species in the sediments of various reservoirs based on LEfSe analysis; Table S1: Mobile phase gradient elution program. Table S2: The primer information of qPCR assays; Table S3: Standard curve of selected genes for qPCR.; Table S4: Physicochemical characteristics of the water samples; Table S5: The Concentration of antimicrobial agents in different water sample sites. (ng/L); Table S6: The Concentration of antimicrobial agents in different sediment sample sites. (ng/g); Table S7: The contents of various heavy metals in reservoir sediments. (mg/kg); Table S8: The α-diversity of the bacterial community. References [11,72,73,74,75,76,77,78,79,80,81,82] are cited in Supplementary Materials.

## Abbreviations

The following abbreviations are used in this manuscript:

ARGs	Antibiotic Resistance Genes
MRGs	Mobile Genetic Elements
HGT	Horizontal Gene Transfer
COND	Conductivity
OPR	Oxidation–Reduction Potential
RC	Residual Chlorine
TDS	Total Dissolved Solids
COD	Chemical Oxygen Demand
TCs	Tetracycline antibiotics
FQs	Fluoroquinolones
EFX	Enrofloxacin

## References

[1] Nnah E.P., Asante J., Amoako D.G., Abia A.L.K., Essack S.Y. (2025). Antibiotic-resistant Escherichia coli (*E. coli*) at one health interfaces in Africa: A scoping review. Sci. Total Environ..

[2] De Briyne N. (2017). One Health perspective of antimicrobial resistance. Vet. Rec..

[3] Mu X., Huang Z., Ohore O.E., Yang J., Peng K., Li S., Li X. (2023). Impact of antibiotics on microbial community in aquatic environment and biodegradation mechanism: A review and bibliometric analysis. Environ. Sci. Pollut. Res..

[4] Guo Z., Kodikara D., Albi L.S., Hatano Y., Chen G., Yoshimura C., Wang J. (2023). Photodegradation of organic micropollutants in aquatic environment: Importance, factors and processes. Water Res..

[5] Wu Z., Shao X., Wang Q. (2025). Antibiotics and Antibiotic Resistance Genes in the Environment: Dissemination, Ecological Risks, and Remediation Approaches. Microorganisms.

[6] Bueno I., He H., Kinsley A.C., Ziemann S.J., Degn L.R., Nault A.J., Beaudoin A.L., Singer R.S., Wammer K.H., Arnold W.A. (2023). Biodegradation, photolysis, and sorption of antibiotics in aquatic environments: A scoping review. Sci. Total Environ..

[7] Ahmed I., Zhang Y., Sun P., Xie Y., Zhang B. (2023). Sensitive response mechanism of ARGs and MGEs to initial designed temperature during swine manure and food waste co-composting. Environ. Res..

[8] Alexander J., Hembach N., Schwartz T. (2022). Identification of critical control points for antibiotic resistance discharge in sewers. Sci. Total Environ..

[9] Hu Y., Yan X., Shen Y., Di M., Wang J. (2018). Antibiotics in surface water and sediments from Hanjiang River, Central China: Occurrence, behavior and risk assessment. Ecotox Environ. Safe.

[10] Zhang K., Fan Y., Chang S., Fu Q., Zhang Q., Yang G., Sun X. (2022). Characterization of antibiotic resistance genes in drinking water sources of the Douhe Reservoir, Tangshan, northern China: The correlation with bacterial communities and environmental factors. Environ. Sci. Eur..

[11] Zhang Z., Yuan W. (2023). Occurrence and distribution of antibiotics and antibiotic resistance genes in the different croplands along the Yellow River shoreline. Environ. Res. Commun..

[12] Swain P.P., Sahoo R.K. (2025). Blocking horizontal transfer of antibiotic resistance genes: An effective strategy in combating antibiotic resistance. Crit. Rev. Microbiol..

[13] Liu F., Luo Y., Xu T., Lin H., Qiu Y., Li B. (2024). Current examining methods and mathematical models of horizontal transfer of antibiotic resistance genes in the environment. Front. Microbiol..

[14] Yamin D., Uskoković V., Wakil A., Goni M., Shamsuddin S., Mustafa F., Alfouzan W., Alissa M., Alshengeti A., Almaghrabi R. (2023). Current and Future Technologies for the Detection of Antibiotic-Resistant Bacteria. Diagnostics.

[15] Wang H.Y., Kim S., Kim J., Park S.D., Kim H.Y., Uh Y., Lee H. (2016). Comparison of multiplex real-time PCR and PCR-reverse blot hybridization assay for the direct and rapid detection of bacteria and antibiotic resistance determinants in positive culture bottles. J. Med. Microbiol..

[16] Dang C., Xia Y., Zheng M., Liu T., Liu W., Chen Q., Ni J. (2020). Metagenomic insights into the profile of antibiotic resistomes in a large drinking water reservoir. Environ. Int..

[17] Li S., Ondon B.S., Ho S.-H., Zhou Q., Li F. (2023). Drinking water sources as hotspots of antibiotic-resistant bacteria (ARB) and antibiotic resistance genes (ARGs): Occurrence, spread, and mitigation strategies. J. Water Process Eng..

[18] Li J., Li L., Li Q., Fang W., Sun Y., Lu Y., Wang J., Zhu Y., Zhang Y. (2023). Distribution and relationship of antibiotics, heavy metals and resistance genes in the upstream of Hanjiang River Basin in Shiyan, China. Environ. Geochem. Health.

[19] Gao F.-Z., Hu L.-X., Liu Y.-S., Yang H.-Y., He L.-Y., Bai H., Liu F., Jin X.-W., Ying G.-G. (2025). Unveiling the prevalence of metal resistance genes and their associations with antibiotic resistance genes in heavy metal-contaminated rivers. Water Res..

[20] Li Y., Chen H., Song L., Wu J., Sun W., Teng Y. (2021). Effects on microbiomes and resistomes and the source-specific ecological risks of heavy metals in the sediments of an urban river. J. Hazard. Mater..

[21] Yang J., Xiang J., Xie Y., Yu K., Li J., Wang H., Li P., Gin K.Y.-H., He Y. (2022). Removal behavior and key drivers of antibiotic resistance genes in two full-scale leachate treatment plants. Water Res..

[22] Sun S., Geng J., Li B., Ma L., Sun X., Meng F., Qi H., Shen J. (2021). Temporal variations of antibiotic resistance genes in influents and effluents of a WWTP in cold regions. J. Clean. Prod..

[23] (2002). Environmental Quality Standards for Surface Water.

[24] Magoc T., Salzberg S.L. (2011). FLASH: Fast length adjustment of short reads to improve genome assemblies. Bioinformatics.

[25] Edgar R.C. (2010). Search and clustering orders of magnitude faster than BLAST. Bioinformatics.

[26] Wang Q., Garrity G.M., Tiedje J.M., Cole J.R. (2007). Naive Bayesian classifier for rapid assignment of rRNA sequences into the new bacterial taxonomy. Appl. Environ. Microbiol..

[27] DeSantis T.Z., Hugenholtz P., Larsen N., Rojas M., Brodie E.L., Keller K., Huber T., Dalevi D., Hu P., Andersen G.L. (2006). Greengenes, a chimera-checked 16S rRNA gene database and workbench compatible with ARB. Appl. Environ. Microbiol..

[28] He L.Y., Ying G.G., Liu Y.S., Su H.C., Chen J., Liu S.S., Zhao J.L. (2016). Discharge of swine wastes risks water quality and food safety: Antibiotics and antibiotic resistance genes from swine sources to the receiving environments. Environ. Int..

[29] Li S., Shi W., You M., Zhang R., Kuang Y., Dang C., Sun W., Zhou Y., Wang W., Ni J. (2019). Antibiotics in water and sediments of Danjiangkou Reservoir, China: Spatiotemporal distribution and indicator screening. Environ. Pollut..

[30] Ariyani M., Jansen L.J.M., Balzer-Rutgers P., Hofstra N., van Oel P., van de Schans M.G.M. (2024). Antibiotic residues in the cirata reservoir, Indonesia and their effect on ecology and the selection for antibiotic-resistant bacteria. Environ. Res..

[31] Zhang L., Liu L., Zhang Y. (2023). Effects of Different Submerged Macrophytes on the Water and Sediment in Aquaculture Ponds with Enrofloxacin Residues. Water.

[32] Zhang Z., Sun K., Gao B., Zhang G., Liu X., Zhao Y. (2011). Adsorption of tetracycline on soil and sediment: Effects of pH and the presence of Cu(II). J. Hazard. Mater..

[33] Zhang Y., Chen H., Jing L., Teng Y. (2020). Ecotoxicological risk assessment and source apportionment of antibiotics in the waters and sediments of a peri-urban river. Sci. Total Environ..

[34] Fu L., Li J., Wang G., Luan Y., Dai W. (2021). Adsorption behavior of organic pollutants on microplastics. Ecotoxicol. Environ. Saf..

[35] Lai H.-T., Lin J.-J. (2009). Degradation of oxolinic acid and flumequine in aquaculture pond waters and sediments. Chemosphere.

[36] Yuan Q., Sui M., Qin C., Zhang H., Sun Y., Luo S., Zhao J. (2022). Migration, Transformation and Removal of Macrolide Antibiotics in The Environment: A Review. Environ. Sci. Pollut. Res..

[37] Guo X., Feng C., Gu E., Tian C., Shen Z. (2019). Spatial distribution, source apportionment and risk assessment of antibiotics in the surface water and sediments of the Yangtze Estuary. Sci. Total Environ..

[38] Zhang T., Sun F., Lei Q., Jiang Z., Luo J., Lindsey S., Xu Y., Liu H. (2022). Quantification of soil element changes in long-term agriculture: A case study in Northeast China. Catena.

[39] Ohore O.E., Addo F.G., Han N., Li X., Zhang S. (2020). Profiles of ARGs and their relationships with antibiotics, metals and environmental parameters in vertical sediment layers of three lakes in China. J. Environ. Manag..

[40] Noulas C., Tziouvalekas M., Karyotis T. (2018). Zinc in soils, water and food crops. J. Trace Elem. Med. Biol..

[41] Mohammed S., Alsafadi K., Hennawi S., Mousavi S.M.N., Kamal-Eddin F.B., Harsanyie E. (2021). Effects of long-term agricultural activities on the availability of heavy metals in Syrian soil: A case study in southern Syria. J. Saudi Soc. Agric. Sci..

[42] Gaze W.H., Zhang L., Abdouslam N.A., Hawkey P.M., Calvo-Bado L., Royle J., Brown H., Davis S., Kay P., Boxall A.B. (2011). Impacts of anthropogenic activity on the ecology of class 1 integrons and integron-associated genes in the environment. ISME J..

[43] Ma L., Li A.D., Yin X.L., Zhang T. (2017). The Prevalence of Integrons as the Carrier of Antibiotic Resistance Genes in Natural and Man-Made Environments. Environ. Sci. Technol..

[44] Liu X., Lv K., Deng C., Yu Z., Shi J., Johnson A.C. (2019). Persistence and migration of tetracycline, sulfonamide, fluoroquinolone, and macrolide antibiotics in streams using a simulated hydrodynamic system. Environ. Pollut..

[45] Xia X., Gu Q., Chen L., Zhang J., Guo W., Liu Z., Li A., Jiang X., Deng M., Zeng J. (2025). Metagenomic assembly insight into the antibiotic resistance genes and antibiotic resistant bacteria in packaged drinking water system. J. Environ. Chem. Eng..

[46] Ben W., Wang J., Cao R., Yang M., Zhang Y., Qiang Z. (2017). Distribution of antibiotic resistance in the effluents of ten municipal wastewater treatment plants in China and the effect of treatment processes. Chemosphere.

[47] Niu Z.-G., Zhang K., Zhang Y. (2016). Occurrence and distribution of antibiotic resistance genes in the coastal area of the Bohai Bay, China. Mar. Pollut. Bull..

[48] Dong P., Cui Q., Fang T., Huang Y., Wang H. (2019). Occurrence of antibiotic resistance genes and bacterial pathogens in water and sediment in urban recreational water. J. Environ. Sci..

[49] Huang Z., Zhao W., Xu T., Zheng B., Yin D. (2019). Occurrence and distribution of antibiotic resistance genes in the water and sediments of Qingcaosha Reservoir, Shanghai, China. Environ. Sci. Eur..

[50] Wang Y., Yang L., Ma J., Tang J., Chen M. (2023). Unraveling the antibiotic resistome in backwater zones of large cascade reservoirs: Co-occurrence patterns, horizontal transfer directions and health risks. J. Environ. Manag..

[51] Mao D., Luo Y., Mathieu J., Wang Q., Feng L., Mu Q., Feng C., Alvarez P.J.J. (2014). Persistence of Extracellular DNA in River Sediment Facilitates Antibiotic Resistance Gene Propagation. Environ. Sci. Technol..

[52] Yang Y., Cui K., Huang Y., Yu K., Li C., Chen Y. (2025). Differential insights into the distribution characteristics of bacterial communities and their response to typical pollutants in the sediment and soil of large drinking water reservoir. J. Environ. Manag..

[53] Guo J., Zheng Y., Teng J., Wang X., Song J. (2021). Characteristics of spatial distribution for microbial ecology inside and outside source water reservoir. J. Clean. Prod..

[54] Ellabaan M.M.H., Munck C., Porse A., Imamovic L., Sommer M.O.A. (2021). Forecasting the dissemination of antibiotic resistance genes across bacterial genomes. Nat. Commun..

[55] Zhang J., Lin H., Ma J., Sun W., Yang Y., Zhang X. (2019). Compost-bulking agents reduce the reservoir of antibiotics and antibiotic resistance genes in manures by modifying bacterial microbiota. Sci. Total Environ..

[56] Lajqi Berisha N., Poceva Panovska A., Hajrulai-Musliu Z. (2024). Antibiotic Resistance and Aquatic Systems: Importance in Public Health. Water.

[57] Qin Y., Tang Q., Lu L., Wang Y., Izaguirre I., Li Z. (2021). Changes in planktonic and sediment bacterial communities under the highly regulated dam in the mid-part of the Three Gorges Reservoir. Appl. Microbiol. Biotechnol..

[58] Chen Y., Liu Y., Wang X. (2017). Spatiotemporal variation of bacterial and archaeal communities in sediments of a drinking reservoir, Beijing, China. Appl. Microbiol. Biotechnol..

[59] Cheng W., Zhang J., Wang Z., Wang M., Xie S. (2014). Bacterial communities in sediments of a drinking water reservoir. Ann. Microbiol..

[60] Li Y., Li R., Hou J., Sun X., Wang Y., Li L., Yang F., Yao Y., An Y. (2024). Mobile genetic elements affect the dissemination of antibiotic resistance genes (ARGs) of clinical importance in the environment. Environ. Res..

[61] Liao X., Chen C., Wang Z., Wan R., Chang C.-H., Zhang X., Xie S. (2013). Changes of biomass and bacterial communities in biological activated carbon filters for drinking water treatment. Process Biochem..

[62] Fishbein S.R.S., Mahmud B., Dantas G. (2023). Antibiotic perturbations to the gut microbiome. Nat. Rev. Microbiol..

[63] Alexandrino D.A.M., Mucha A.P., Almeida C.M.R., Gao W., Jia Z., Carvalho M.F. (2017). Biodegradation of the veterinary antibiotics enrofloxacin and ceftiofur and associated microbial community dynamics. Sci. Total Environ..

[64] Kayal A., Mandal S. (2022). Microbial degradation of antibiotic: Future possibility of mitigating antibiotic pollution. Environ. Monit. Assess..

[65] Yang C.-W., Hsiao W.-C., Chang B.-V. (2016). Biodegradation of sulfonamide antibiotics in sludge. Chemosphere.

[66] He L.-X., He L.-Y., Gao F.-Z., Zhang M., Chen J., Jia W.-L., Ye P., Jia Y.-W., Hong B., Liu S.-S. (2023). Mariculture affects antibiotic resistome and microbiome in the coastal environment. J. Hazard. Mater..

[67] Han C., Cao H., Tan H., Li X., Yang W. (2024). Distribution and community structure of antibiotic resistance genes in the Three Gorges Reservoir Area. Environ. Sci. Pollut. Res..

[68] Zhou Z.C., Feng W.Q., Han Y., Zheng J., Chen T., Wei Y.Y., Gillings M., Zhu Y.G., Chen H. (2018). Prevalence and transmission of antibiotic resistance and microbiota between humans and water environments. Environ. Int..

[69] Gao M., Qiu T., Sun Y., Wang X. (2018). The abundance and diversity of antibiotic resistance genes in the atmospheric environment of composting plants. Environ. Int..

[70] Zhao H., Zhang J., Chen X., Yang S., Huang H., Pan L., Huang L., Jiang G., Tang J., Xu Q. (2023). Climate and nutrients regulate biographical patterns and health risks of antibiotic resistance genes in mangrove environment. Sci. Total Environ..

[71] Zhang Z., Zhang Q., Wang T., Xu N., Lu T., Hong W., Penuelas J., Gillings M., Wang M., Gao W. (2022). Assessment of global health risk of antibiotic resistance genes. Nat. Commun..

[72] Contreras P.J., Urrutia H., Sossa K., Nocker A. (2011). Effect of PCR amplicon length on suppressing signals from membrane-compromised cells by propidium monoazide treatment. Microbiol. Methods.

[73] Goldstein C., Lee Margie D., Sanchez S., Hudson C., Phillips B., Register B., Grady M., Liebert C., Summers Anne O., White David G. (2001). Incidence of Class 1 and 2 Integrases in Clinical and Commensal Bacteria from Livestock, Companion Animals, and Exotics. Antimicrob. Agents Chemother..

[74] Wang L., Zhao X., Wang J., Wang J., Zhu L., Ge W. (2019). Macrolide- and quinolone-resistant bacteria and resistance genes as indicators of antibiotic resistance gene contamination in farmland soil with manure application. Ecol. Indic..

[75] Xia R., Guo X., Zhang Y., Xu H. (2010). qnrVC-Like Gene Located in a Novel Complex Class 1 Integron Harboring the ISCR1 Element in an Aeromonas punctata Strain from an Aquatic Environment in Shandong Province, China. Antimicrob. Agents Chemother..

[76] Heuer H., Smalla K. (2007). Manure and sulfadiazine synergistically increased bacterial antibiotic resistance in soil over at least two months. Environ. Microbiol..

[77] Alneama R.T., Al-Massody A.J., Mahmud B.M., Ghasemian A. (2021). The existence and expression of aminoglycoside resistance genes among multidrug-resistant Escherichia coli isolates in intensive care unit centers. Gene Rep..

[78] Xu Y., Gao H., Li R., Lou Y., Li B., Cheng G., Na G. (2024). Occurrence and distribution of antibiotics and antibiotic resistance genes from the land to ocean in Daliao River-Liaodong Bay, China. Mar. Environ. Res..

[79] Zhang K., Gu J., Wang X., Zhang X., Hu T., Zhao W. (2019). Analysis for microbial denitrification and antibiotic resistance during anaerobic digestion of cattle manure containing antibiotic. Bioresour. Technol..

[80] Li Y., Zhao J., Li Y., Jin B., Zhang K., Zhang H. (2020). Long-term alkaline conditions inhibit the relative abundances of tetracycline resistance genes in saline 4-chlorophenol wastewater treatment. Bioresour. Technol..

[81] Shen X., Jin G., Zhao Y., Shao X. (2020). Prevalence and distribution analysis of antibiotic resistance genes in a large-scale aquaculture environment. Sci. Total Environ..

[82] Ni B.-J., Zeng S., Wei W., Dai X., Sun J. (2020). Impact of roxithromycin on waste activated sludge anaerobic digestion: Methane production, carbon transformation and antibiotic resistance genes. Sci. Total Environ..

