# Supplementary material for: Occurrence and Distribution of Antibiotics and Antibiotic Resistance Genes in the Water and Sediments of Reservoir-Based Drinking Water Sources in Henan, China
Source: Microorganisms. 2025 Dec 12;13(12):2828. doi: 10.3390/microorganisms13122828 (PMC12735494; doi:10.3390/microorganisms13122828)
Supplement: Supplementary file 1 [file microorganisms-13-02828-s001.zip › microorganisms-4012701-supplementary.pdf]

Supporting materials

List

Text S1. Detailed design of the sampling methodology

Text S2. The TQ-LC-MS analysis of the antibiotics

Text S3. The ICP-OES analysis of the heavy metals

Figure S1. Geographical distribution of sampling points in reservoir-type water source area.

Figure S2. Distribution of the top 50 differentially abundant species in the water bodies of various reservoirs based on LEfSe analysis.

Figure S3. Distribution of the top 50 differentially abundant species in the sediments of various reservoirs based on LEfSe analysis.

Table S1. Mobile phase gradient elution program.

Table S2. The primer information of qPCR assays.

Table S3. Standard curve of selected genes for qPCR.

Table S4. Physicochemical characteristics of the water samples.

Table S5. The Concentration of antimicrobial agents in different water sample sites. (ng/L)

Table S6. The Concentration of antimicrobial agents in different sediment sample sites. (ng/g)

Table S7. The contents of various heavy metals in reservoir sediments. (mg/kg)

Table S8. The α-diversity of bacterial community.

Text S1. Detailed design of the sampling methodology

Taking the LQ Reservoir as an example, the sampling points were deployed following a "point-belt" coupled gradient design: one main cross-section was established at both the inlet and the outlet. Using the "point-section" metho, a central sampling point was precisely positioned. Along the main cross-section in the flow direction, starting from the central point, the sampling belt was extended 50 m upstream and 50 m downstream respectively, forming a 100-m-long belt-shaped section and thus a "1 center + 2 gradients" three-point array. Within each array, water samples from the three points were collected in equal volumes (1 L each) and mixed immediately to produce a composite representative sample for that cross-section, yielding the inlet composite sample LQ1 and the outlet composite sample LQ2. The other reservoirs (JG and HGK) followed the same spatial layout to ensure comparability of sampling points and gradient consistency across different reservoirs.

Text S2. The TQ-LC-MS analysis of the antibiotics

Antibiotic concentrations were determined with a Triple Quad™ 3500 TQ-LC-MS system (SCIEX). Mobile phases were (A) ultrapure water containing 0.1 % formic acid and (B) acetonitrile containing 0.1 % formic acid, delivered by the gradient programme listed in Table X. Mass-spectrometric conditions were: SRM scan mode; transfer-tube temperature 325 °C; source temperature 350 °C; electrospray voltage (+) 4.0 kV; auxiliary gas 10 arb; sheath gas 40 arb; collision gas 2 mTorr.

Text S3. The ICP-OES analysis of the heavy metals

Sediment samples were first cleared of visible debris, lyophilized, and ground in an acid-washed agate mortar to pass a 100-mesh nylon sieve. Exactly 0.3000 g of the homogenized powder was transferred into a 50 mL PTFE digestion vessel; triplicate subsamples and one method blank were prepared in parallel. The powder was moistened with 2 mL ultrapure water, followed by the sequential addition of 10 mL suprapur HNO_3, 5 mL HF, and 2 mL $HClO_4$. Vessels were then loaded into an automated graphite digestion block programmed as follows: 60 °C for 30 min, ramped to 120 °C

for 60 min, raised to 160 °C for 90 min, and finally held at 180 °C for 40 min. Digestion was deemed complete when the solution appeared clear and colorless to pale yellow with no visible residue or fumes. After cooling to ambient temperature, the digest was quantitatively transferred into a 25 mL volumetric flask with 2 % HNO_3 (v/v, prepared from suprapur acid and ultrapure water), rinsing the vessel three times to ensure quantitative recovery. The flask was made up to volume with 2 % HNO_3, tightly sealed, and homogenized at 2000 rpm for 2 min on a vortex mixer. The final solution was transferred to a centrifuge tube and spun at 4000 rpm for 15 min before analysis.

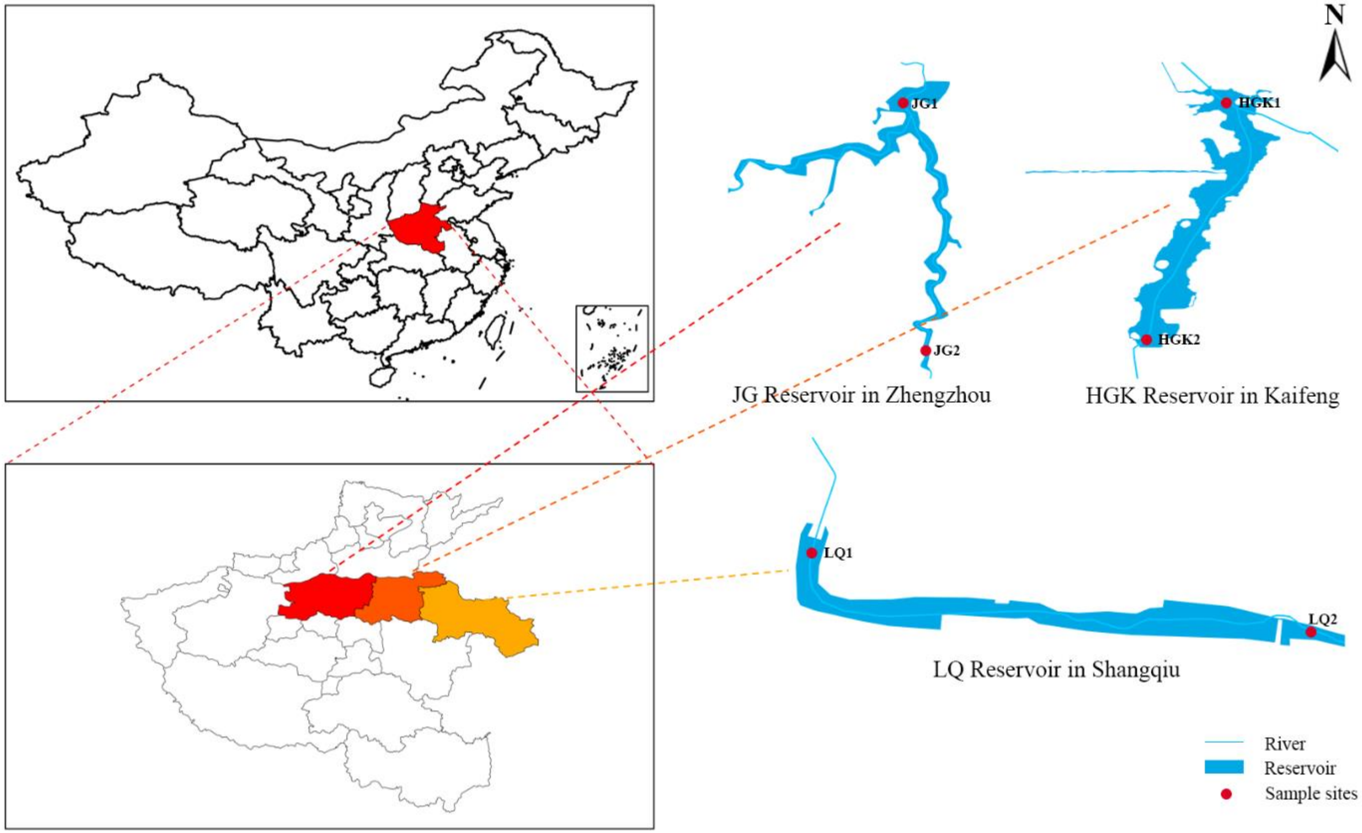

Figure S1. Geographical distribution of sampling points in reservoir-type water source area.

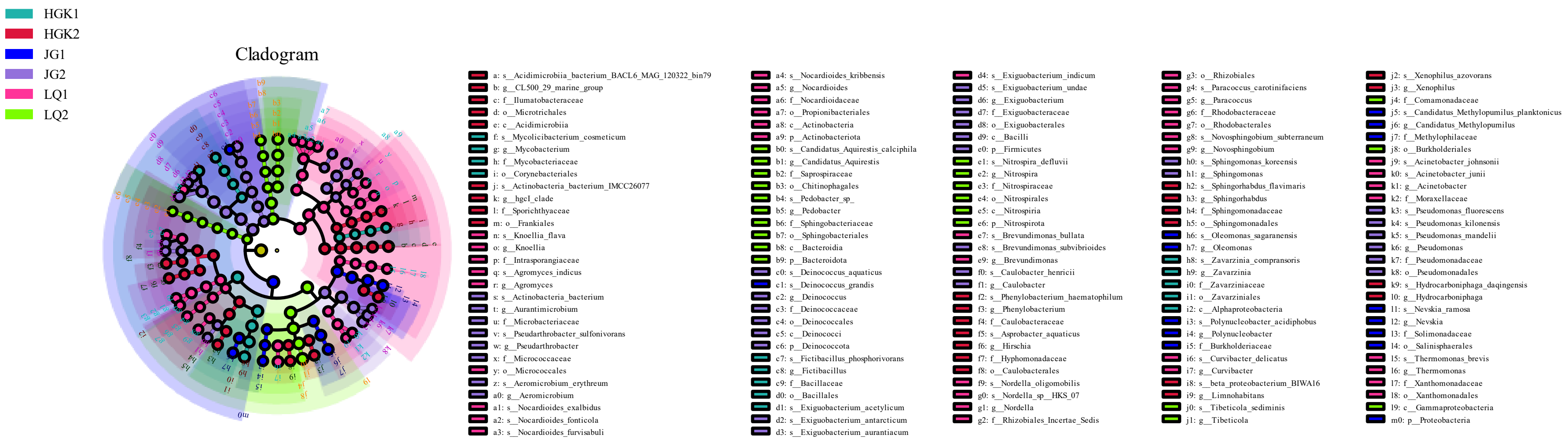

Figure S2. Distribution of the top 50 differentially abundant species in the water bodies of various reservoirs based on LEfSe analysis.

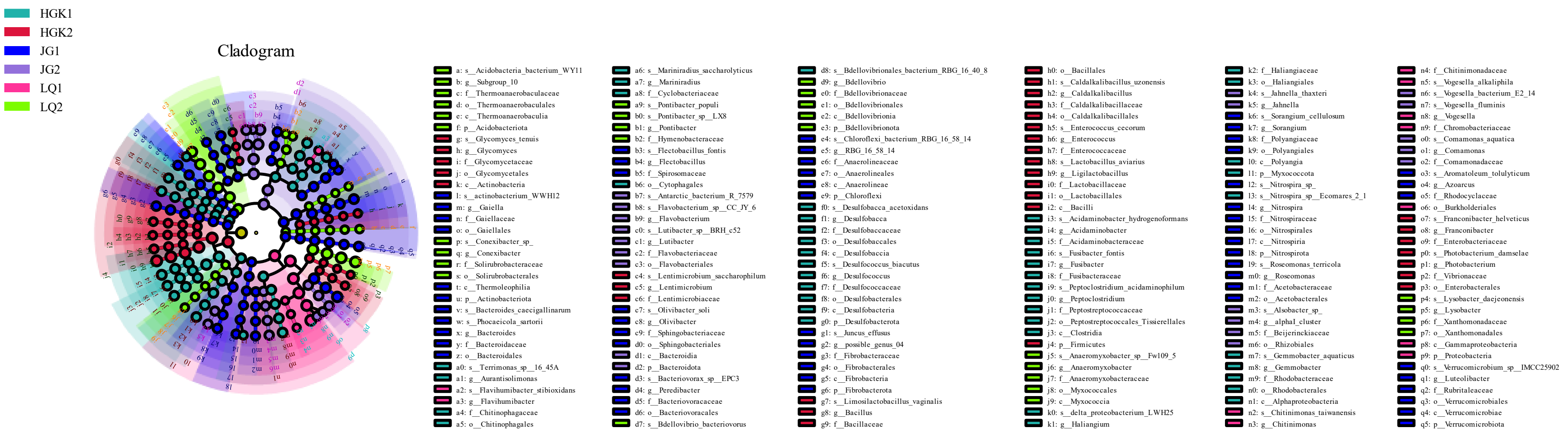

Figure S3. Distribution of the top 50 differentially abundant species in the sediments of various reservoirs based on LEfSe analysis.

Table S1. Mobile phase gradient elution program.

Time (min)	A (%)	B (%)	Flow rate (mL / min)
0.00	95	5	0.3
2.00	85	15	0.3
4.00	75	15	0.3
6.00	75	25	0.3
7.00	5	95	0.3
7.10	95	5	0.3
9.00	95	5	0.3

Table S2. The primer information of qPCR assays.

Targeted genes	Primer sequences (5'-3')	Classification	Annealing temperature (℃)	References
16S rRNA	FW: GAGTTTGATCCTGGCTCAG<br>RV: ATTACCGCGGCTGCTGG	Bacteria	60	[1]
intI1	FW: CCTCCCGCACGATGATC<br>RV: TCCACGCATCGTCAGGC	Integron	60	[2]
intI2	FW: GTTATTTTATTGCTGGGATTAGGC<br>RV: TTTTACGCTGCTGTATGGTGC	Integron	55	[3]
ISCR 1	FW: CGCCCACTCAAACAAACG<br>RV: GAGGCTTTGGTGTAACCG	Transposase	52	[4]
sul1	FW: CCGTTGGCCTTCCTGTAAAG<br>RV: TTGCCGATCGCGTGAAGT	Sulfonamides	60	[5]
aac(6')-Ib	FW: GTTTGAGAGGCAAGGTACCGTAA<br>RV: GAATGCCTGGCGTGTTTGA	Aminoglycosides	55	[6]
aadA	FW: CGAGATTCTCCGCGCTGTA<br>RV: GCTGCCATTCTCCAAATTGC	Aminoglycosides	54	[7]
ermB	FW: GAGATCGGRCCAGGAAGC<br>RV: GTGTGCACCATCGCCTGA	Macrolides	61	[8]
tetA	FW: GCTGTTTGTTCTGCCGGAAA<br>RV: GGTTAAGTTCCTTGAACGCAAACT	Tetracyclines	54	[9]
tetX	FW: AGCCTTACCAATGGGTGTAAA<br>RV: TTCTTACCTTGGACATCCCG	Tetracyclines	60	[10]
$bla_{CTX\text{-}M}$	FW: GGAGGCGTGACGGCTTTT<br>RV: TTCAGTGCGATCCAGACGAA	β-lactams	56	[11]
qnrA	FW: TTACTGCATCCATACGCTGCTT<br>RV: ATTTCTCACGCCAGGATTTG	Quinolones	54	[12]

Table S3. Standard curve of selected genes for qPCR.

**Genes**	**Standard curve**	**R^2**
16S rRNA	y=-3.407x+36.00	0.995
intI1	y=-3.029x+33.79	0.993
intI2	y=-3.093x+33.61	0.995
ISCR 1	y=-3.110x+39.33	0.999
sul1	y=-2.932x+34.14	0.990
aac(6')-Ib	y=-2.974x+34.705	0.996
aadA	y=-3.0826x+35.188	0.998
ermB	y= -2.928x+32.48	0.991
tetA	y=-3.6009x+42.309	0.991
tetX	y=-3.046x+34.63	0.995
$bla_{CTX\text{-}M}$	y=-2.7213x+33.879	0.996
qnrA	y=-2.839x+33.23	0.999

Table S4. Physicochemical characteristics of the water samples.

Samples	COND (μs·cm^{-1})	TDS (mg/L)	ORP (mv)	pH	RC (mg/L)	COD (mg/L)
**LQ1**	641.00 ± 0.36	436.50 ± 0.26	210.67 ± 3.51	7.44 ± 0.04	0.02 ± 0.00	169.84 ± 0.69
**LQ2**	810.00 ± 0.17	556.07 ± 0.35	79.33 ± 4.04	7.08 ± 0.07	0.02 ± 0.00	123.37 ± 0.37
**JG1**	200.03 ± 0.57	130.57 ± 0.61	169.67 ± 4.51	7.58 ± 0.06	0.01 ± 0.00	3.66 ± 0.52
**JG2**	305.60 ± 0.60	201.60 ± 0.40	193.33 ± 1.15	7.26 ± 0.02	0.02 ± 0.01	3.55 ± 0.57
**HGK1**	760.50 ± 0.26	519.23 ± 0.12	253.00 ± 3.61	7.43 ± 0.00	0.03 ± 0.01	14.51 ± 0.70
**HGK2**	698.17 ± 0.06	475.07 ± 0.15	217.33 ± 5.51	7.37 ± 0.01	0.02 ± 0.00	9.30 ± 0.08

Table S5. The Concentration of antimicrobial agents in different water sample sites. (ng/L)

	TC	CTC	OTC	EFX	LFX	AZI	CLA	ROX	SDZ	SMX	SPD	TMP
**LQ1**	ND	10.41 ± 1.24	ND	107.71 ± 13.55	8.32 ± 0.68	ND	ND	ND	0.34 ± 0.09	0.42 ± 0.08	0.19 ± 0.02	0.41 ± 0.36
**LQ2**	ND	2.12 ± 1.84	ND	3.36 ± 0.15	2.11 ± 0.06	ND	ND	ND	0.14 ± 0.00	0.15 ± 0.15	ND	ND
**JG1**	ND	5.21 ± 0.21	ND	13.87 ± 1.07	5.11 ± 0.98	2.08 ± 0.06	ND	1.91 ± 0.54	0.33 ± 0.05	3.79 ± 0.28	1.07 ± 0.06	ND
**JG2**	ND	5.27 ± 0.3	ND	41.67 ± 1.25	2.78 ± 0.15	2.01 ± 0.02	ND	0.16 ± 0.02	0.22 ± 0.19	3.13 ± 0.11	0.23 ± 0.05	ND
**HGK1**	ND	8.25 ± 0.01	ND	96.58 ± 3.87	16.57 ± 1.97	ND	ND	ND	0.41 ± 0.07	0.32 ± 0.06	0.22 ± 0.05	0.63 ± 0.05
**HGK2**	ND	8.15 ± 0.36	ND	20.98 ± 4.94	3.08 ± 0.19	2.11 ± 0.04	0.08 ± 0.04	1.71 ± 0.05	0.54 ± 0.03	1.98 ± 0.08	0.23 ± 0.02	ND

ND - Not Detected.

Table S6. The Concentration of antimicrobial agents in different sediment sample sites. (ng/g)

Samples	TC	CTC	OTC	EFX	LFX	NFX	AZI	CLA	ROX	SDZ	SMX	SPD	TMP
**LQ1**	1.41 ± 0.00	1.33 ± 0.10	ND	2.48 ± 0.57	9.30 ± 0.36	12.35 ± 0.00	ND	ND	ND	ND	0.88 ± 0.06	0.27 ± 0.38	ND
**LQ2**	1.41 ± 0.00	5.47 ± 0.87	ND	2.76 ± 0.69	8.81 ± 0.26	9.55 ± 4.01	ND	ND	ND	0.13 ± 0.04	1.23 ± 0.13	0.28 ± 0.39	ND
**JG1**	1.41 ± 0.00	1.23 ± 0.04	ND	2.66 ± 0.75	8.57 ± 0.11	10.39 ± 2.18	ND	ND	ND	ND	0.82 ± 0.04	0.56 ± 0.04	ND
**JG2**	1.41 ± 0.00	1.28 ± 0.08	ND	3.80 ± 1.26	8.74 ± 0.32	11.43 ± 2.02	14.23 ± 2.06	ND	0.85 ± 0.30	ND	0.98 ± 0.13	ND	ND
**HGK1**	1.41 ± 0.00	1.34 ± 0.00	ND	4.71 ± 3.97	8.54 ± 0.00	12.22 ± 0.00	2.09 ± 0.03	ND	0.31 ± 0.00	0.13 ± 0.06	0.86 ± 0.07	0.27 ± 0.38	ND
**HGK2**	1.42 ± 0.01	1.43 ± 0.08	1.36± 0.00	3.16 ± 0.55	8.71 ± 0.06	12.99 ± 0.54	3.58 ± 0.31	ND	0.48 ± 0.27	0.11 ± 0.00	0.87 ± 0.10	0.28 ± 0.39	ND

ND - Not Detected.

Table S7. The contents of various heavy metals in reservoir sediments. (mg/kg)

Samples	As	Zn	Cu	Ni	Co	Fe	Mn	Cr	V	Ti	Pb	Cd
**LQ1**	21.58	47.27	16.86	61.16	9.94	3.56	498.03	84.91	87.67	3174.35	19.18	0.33
**LQ2**	5.91	42.37	14.84	46.25	9.29	3.34	394.88	79.25	85.62	3195.25	18.59	0.22
**JG1**	5.53	35.31	15.03	54.18	9.16	3.04	396.14	87.42	93.93	3418.43	17.87	0.21
**JG2**	126.10	362.03	12.80	47.47	7.57	2.43	340.18	65.59	68.62	2634.96	13.16	0.38
**HGK1**	7.94	31.21	10.50	40.73	7.18	2.43	361.14	76.78	75.70	2554.07	18.32	0.25
**HGK2**	17.75	121.46	21.37	61.68	9.72	3.52	487.57	85.26	89.49	3340.63	20.38	0.27

Table S8. The α-diversity of bacterial community.

Samples		Feature	Richness		Diversity		PD_whole_tree	Coverage
			Ace	Chao1	Simpson	Shannon		
**Water**	LQ1	576	576.8111	576.2308	0.9784	7.2253	28.3293	1
	LQ2	447	448.7264	448.6667	0.9813	7.1654	31.5776	0.9999
	JG1	442	444.1312	444	0.9751	6.5912	30.0313	0.9999
	JG2	330	334.4735	337.8	0.9489	5.6438	18.6078	0.9997
	HGK1	570	570.5604	570.5	0.9839	7.4823	32.0197	0.9999
	HGK2	485	486.3482	486.5	0.9806	7.0332	32.2916	0.9999
**Sediment**	LQ1	2587	2594.577	2587.483	0.9955	9.5079	289.9506	0.9996
	LQ2	3636	3652.365	3637.512	0.995	10.2251	432.5825	0.9992
	JG1	3636	3653.823	3637.51	0.9966	10.1342	428.3373	0.9991
	JG2	3070	3081.749	3070.64	0.9975	10.068	275.9337	0.9994
	HGK1	3592	3614.113	3593.965	0.9985	10.4725	370.8089	0.9989
	HGK2	5297	5303.027	5297.106	0.9938	10.564	650.2304	0.9997

Reference

1. Contreras P.J.;Urrutia H.;Sossa K.;Nocker A. Effect of PCR amplicon length on suppressing signals from membrane-compromised cells by propidium monoazide treatment. *Microbiol Methods*. **2011**,87,89-95.
2. Goldstein C.;Lee Margie D.;Sanchez S.;Hudson C.;Phillips B.;Register B.;Grady M.;Liebert C.;Summers Anne O.;White David G., et al. Incidence of Class 1 and 2 Integrases in Clinical and Commensal Bacteria from Livestock, Companion Animals, and Exotics. *Antimicrob Agents Ch*. **2001**,45,723-726.
3. Wang L.;Zhao X.;Wang J.;Wang J.;Zhu L.;Ge W. Macrolide- and quinolone-resistant bacteria and resistance genes as indicators of antibiotic resistance gene contamination in farmland soil with manure application. *Ecol Indic*. **2019**,106,105456.
4. Xia R.;Guo X.;Zhang Y.;Xu H. qnrVC-Like Gene Located in a Novel Complex Class 1 Integron Harboring the ISCR1 Element in an Aeromonas punctata Strain from an Aquatic Environment in Shandong Province, China. *Antimicrob Agents Ch*. **2010**,54,3471-3474.
5. Heuer H.;Smalla K. Manure and sulfadiazine synergistically increased bacterial antibiotic resistance in soil over at least two months. *Environ Microbiol*. **2007**,9,657-666.
6. Alneama R.T.;Al-Massody A.J.;Mahmud B.M.;Ghasemian A. The existence and expression of aminoglycoside resistance genes among multidrug-resistant Escherichia coli isolates in intensive care unit centers. *Gene Rep*. **2021**,25,101315.
7. Xu Y.;Gao H.;Li R.;Lou Y.;Li B.;Cheng G.;Na G. Occurrence and distribution of antibiotics and antibiotic resistance genes from the land to ocean in Daliao River-Liaodong Bay, China. *Mar Environ Res*. **2024**,197,106470.
8. Zhang K.;Gu J.;Wang X.;Zhang X.;Hu T.;Zhao W. Analysis for microbial denitrification and antibiotic resistance during anaerobic digestion of cattle manure containing antibiotic. *Bioresour Technol*. **2019**,291,
9. Li Y.;Zhao J.;Li Y.;Jin B.;Zhang K.;Zhang H. Long-term alkaline conditions inhibit the relative abundances of tetracycline resistance genes in saline 4-chlorophenol wastewater treatment. *Bioresour Technol*. **2020**,301,122792.
10. Shen X.;Jin G.;Zhao Y.;Shao X. Prevalence and distribution analysis of antibiotic resistance genes in a large-scale aquaculture environment. *Sci Total Environ*. **2020**,711,134626.
11. Zhang Z.;Yuan W. Occurrence and distribution of antibiotics and antibiotic resistance genes in the different croplands along the Yellow River shoreline. *Environ Res Commun*. **2023**,5,
12. Ni B.-J.;Zeng S.;Wei W.;Dai X.;Sun J. Impact of roxithromycin on waste activated sludge anaerobic digestion: Methane production, carbon transformation and antibiotic resistance genes. *Sci Total Environ*. **2020**,703,134899.
